# Supplementary material for: PlaceWaters: Real-time, explicit interface water sampling during Rosetta ligand docking
Source: PLoS One. 2022 May 31;17(5):e0269072. doi: 10.1371/journal.pone.0269072 (PMC9154094; doi:10.1371/journal.pone.0269072)
Supplement: Attachment — Submitted filename: Response to Reviewers.docx [file pone.0269072.s002.docx]

Dear L. Michel Espinoza-Fonseca,

We have carefully analyzed the reviewer comments and revised our manuscript entitled *RosettaWaters: explicit water sampling during Rosetta ligand docking* following their suggestions. We feel that the manuscript substantially improved. We hope that you will find this revised version acceptable for publication in *Plos One*. Please find a detailed summary of all changes applied to the manuscript and our point-by-point responses to the reviewer comments below.

Best regards,

Shannon Smith

Journal Requirements:

When submitting your revision, we need you to address these additional requirements.

1. Please ensure that your manuscript meets PLOS ONE's style requirements, including those for file naming. The PLOS ONE style templates can be found at 
https://journals.plos.org/plosone/s/file?id=wjVg/PLOSOne_formatting_sample_main_body.pdf and https://journals.plos.org/plosone/s/file?id=ba62/PLOSOne_formatting_sample_title_authors_affiliations.pdf

Line 237 on page 11: changed Figure 6 to Fig 5.

2. We note that you have referenced (Carlson H, Smith R, Damm-Ganamet† K, Stuckey J, Ahmed A, Convery M. CSAR 2014: A Benchmark Exercise Using Unpublished Data from Pharma. Journal of Chemical Information and Modeling. 2016:56(6):1063–7) which has currently not yet been accepted for publication. Please remove this from your References and amend this to state in the body of your manuscript: (Carlson H, Smith R, Damm-Ganamet† K, Stuckey J, Ahmed A, Convery M. CSAR 2014: A Benchmark Exercise Using Unpublished Data from Pharma. Journal of Chemical Information and Modeling. 2016:56(6):1063–7 [Unpublished]”) as detailed online in our guide for authors
<http://journals.plos.org/plosone/s/submission-guidelines#loc-reference-style>

Thank you for observing this. This paper has been published and accepted at Journal of Chemical Information and Modeling. The word “Unpublished” in the title refers to the benchmark dataset used in their publication.

3. Please include captions for your Supporting Information files at the end of your manuscript, and update any in-text citations to match accordingly. Please see our Supporting Information guidelines for more information: <http://journals.plos.org/plosone/s/supporting-information>.

Added “S1 File: Protocol Capture.”

4. Please review your reference list to ensure that it is complete and correct. If you have cited papers that have been retracted, please include the rationale for doing so in the manuscript text, or remove these references and replace them with relevant current references. Any changes to the reference list should be mentioned in the rebuttal letter that accompanies your revised manuscript. If you need to cite a retracted article, indicate the article’s retracted status in the References list and also include a citation and full reference for the retraction notice.

See above #2 for explanation on reference. We have also added the following reference: Kung PP, Huang B, Zhang G, Zhou JZ, Wang J, Digits JA, et al. Dihydroxyphenylisoindoline amides as orally bioavailable inhibitors of the heat shock protein 90 (hsp90) molecular chaperone. J Med Chem. 2010;53(1):499-503. doi: 10.1021/jm901209q. PubMed PMID: 19908836.

Reviewer #1: In this work, the authors introduced a new algorithm to predict the location of bridging waters during molecular docking. This approach is quite exciting and provides relevant information to this highly complex topic. I have just a few comments and suggestions that I would like the authors to address.

- The images in Figure 1 do not provide enough information on their own about the methodology. In addition, the predicted bridging waters (small gray asterisks) are not visible, making it difficult to compare them with the structural water molecules (red spheres). From my point of view, the authors should merge Figure 1 with Figure 2, explaining in more detail the steps of adding bridging waters so that the reader can have a better appreciation of the algorithm.

Thank you for your input on this. We have merged Figures 1 and 2 to better. We hope this better outlines the individual steps of the PlaceWaters step while placing this in the context of the complete docking protocol and in comparison to the other workflows. Figure numbering has been adjusted accordingly.

- I think it is essential to include a case study. It could be the one used in the "provided_protocol" directory.

This is a great suggestion, and we have added the following section in the Results to include this. We have also included a new reference where this structure was published.

## Case-study: Heat shock protein 90 in complex with known inhibitor

For a detailed walk-through of the algorithm, we present a case-study outlining the results from the co-crystal structure of heat shock protein 90 (Hsp90) in complex with a known inhibitor, 4-chloro-6-{[(2R)-2-(2-methylphenyl)pyrrolidin-1-yl]carbonyl}benzene-1,3-diol (PDB ID: 3K97, ligand ID: 4CD) [24]. As can be seen in Fig 1, there are 2 co-crystallized bridging waters: HOH 237, which forms hydrogen bonds with the ligand para-hydroxyl, the L63 backbone carbonyl oxygen and the S67 sidechain hydroxyl group; and HOH 238, which forms hydrogen bonds with the ligand ortho-hydroxyl and ligand amide oxygen, D108 sidechain carboxyl group and the G112 backbone amide nitrogen. All numbering is based on the deposited PDB 3K97.

As can be seen in Fig 1A, the initial grid successfully identifies the regions where HOH 237 and 238 were co-crystallized, as well as a large region on the solvent-facing side of the ligand. Fig1B shows the results from the clustering and merging step resulting in 6 waters being placed around the ligand, including waters placed at 1.2Å and 0.3Å of HOH 237 and 238, respectively. As shown in Fig1C, the PlaceWaters mover kept the water molecule near the HOH 238, and successfully recaptured this hydrogen bond network. The water placed close to HOH 237; however, was removed during the final selection step due to being too far from either a ligand or protein hydrogen bond donor or acceptor atom.

We also added several sentences in the Discussion that discuss the findings from the case-study in the context of parameter optimization.

As seen in the 3K97 case-study, the final selection step removed a bridging water molecule near HOH 237 due to being slightly too far from a hydrogen bond donor or acceptor atom. In this case, it would be better to tune these distance settings. Another potential way to improve this is to do a more fine-grained sampling of the water within a defined sphere to check for nearby hydrogen bond acceptor and donor atoms. This could avoid such sharp cutoffs and be less reliant on the coarse-grained merged coordinate represented.

- Page 17, Line 226. Reference error.

Corrected--changed Figure 6 to Fig 5.

- When I tried to use the provided protocol scripts, I ran into some issues:
(1) Authors should specify the packages from the beginning that should be installed, i.e., Corina, OpenBabel, BCL (Biology and Chemistry Library Project), and Rosetta.

The following lines have been added to the top of the README.md file in Supplementary Materials.

\# Version information for installed software:

\### All protocols are run on Rosetta master v3.13 in GitHub branch laurashub/place_waters. This will be in the upcoming Rosetta distributed version.

\### BCL (Biology and Chemistry Library) v4.2.0

\### Corina 4.3.0 (10/30/2019)

\### OpenBabel v2.4.1 (3/20/2017)

(2) Is there any additional option to prepare the 3D structures of the ligands instead of Corina? In my case I used OpenBabel (babel -isdf ${prefix}.native.sdf -osdf ${prefix}.corina.sdf --gen3D).

OpenBabel can be used for this step, as long as the --gen3D flag is provided as you have here.
(3) There seems to be an error in the PARAMS file. The 3-letter code should be 4CD instead of 3K9; otherwise, the program throws an error.

Thank you for catching this. Yes the ligand name is supposed to be 4CD to match with the PDB file. The prep_inputfiles.sh shows this change in the molfiletoparams line with the -n option set to ${lig}.

(4) Will a particular version or plug-in of Rosetta should be installed/included to run this module? I tried to run it with my actual version and got the following error: "Element 'PlaceWaters': This element is not expected". I think it's important to let the reader know where they can find this information (e.g., github).

The ‘PlaceWaters’ mover is being worked into the Rosetta version to be released next, but is not yet in the currently distributed package (as of April 14, 2022). A working version of this can currently be cloned, compiled and run from the RosettaCommons GitHub in the laurashub/place_waters branch.
